# Sequential immunizations with a panel of HIV-1 Env virus-like particles coach immune system to make broadly neutralizing antibodies

**DOI:** 10.1038/s41598-018-25960-1

**Published:** 2018-05-17

**Authors:** Teena Mohan, Zachary Berman, Sang-Moo Kang, Bao-Zhong Wang

**Affiliations:** Center for Inflammation, Immunity & Infection, Institute for Biomedical Sciences, Georgia State University, 100 Piedmont Ave SE, Atlanta, GA 30303 USA

## Abstract

Broadly neutralizing antibodies (bnAbs) are correlated with passive HIV/SHIV protection and are desirable components of a HIV protective immunity. In the current study, we have designed a sequential-immunization strategy with a panel of envelope glycoprotein (Env)-enriched virus-like particles (VLPs) from various HIV-1 clades (A-E) to elicit bnAbs with high breadth and potency of neutralization in rabbits. We have compared this regimen with repetitive immunizations of individual Env (subtype B) VLPs or a mixture of various Env VLPs. Our results demonstrate that the sequential immunization group of animals induced significantly higher IgG endpoint titers against respective HIV Env (autologous) antigen than other control groups. Animals vaccinated sequentially showed an increase in the antibody endpoint titers and IgG antibody secreting cells (ASCs) against Con-S Env protein. Sequential immunizations with various Env VLPs promoted antibody avidity indices and enhanced bnAb responses against a panel of HIV pseudotyped virions including some of the tier 3 pseudostrains. Sequential immunizations with various VLPs displaying “native-like” HIV-1 Envs elicited bnAb responses with increased breadth and potency of neutralization.

## Introduction

Human immunodeficiency virus type-1 (HIV-1) has infected near 37 million people globally^1^. A successful HIV vaccine would have a massive influence in curtailing new infections^2^. Although some important progress has been achieved in past three decades, including the RV144 trial which showed an unprecedented 31.2% reduction in HIV incidence, a potentially licensable vaccine candidate remains elusive^3^. Recently, a further endeavor, the HVTN 100 trial has been carried out to evaluate the adapted versions of the RV144 trial designed specifically for the population of South Africa^4^. If several key immune response targets are met, it could set the stage for a far larger Phase III efficacy trial (HVTN 702) with the potential to lead to licensure^5^.

HIV-1 evolves rapidly within the host, resulting in the accumulation of diverse HIV-1 quasispecies^6^. The Env, a virally encoded protein which hides conserved CD4 and co-receptor binding sites with an evolving shield of glycans, variable immunodominant loops, and conformational masks, is the only target for antibodies to neutralize^7^. Though Env presents a moving target to the host immune system, many attempts to generate bnAbs using HIV-1 Env have uniformly failed^8^. This lack of neutralization may arise from the use of monomeric proteins which present epitopes that are not exposed on the “native-like” viral spike. Also, previous vaccination approaches may not successfully display some conserved epitopes that are weakly-immunogenic but critical determinants for bnAbs, to the host immune system^9^.

Although bnAbs are recognized as the “holy grail” of a protective immunity, no HIV vaccine candidate has been able to induce this response. Some HIV infected individuals are found to generate bnAbs after a long period sometimes as long as 2–4 years of infection. These bnAbs are formed through successive cycles of antibody mutation, selection, and virus escape. This process usually takes too long to offer any natural resistance to infection^10^. Although these bnAbs do not help infected individuals to control the virus, they are thought to provide protection when they are in the host immune system prior to an exposure^5^. As the development of bnAbs usually requires extensive antigen exposure over a longperiod, a vaccination strategy should start with an immunogen that presents a specific conserved epitope and then boost the response with the same epitope on a different immunogen to achieve high-affinity recognition of the epitope in the context of the native viral spike^11^. Continuous exposure to the constantly mutating virus can stimulate multiple processes which eventually give rise to potent antibodies capable of neutralizing a wide swath of HIV-1 variants. Thus, it is reasonable to postulate that sequential immunizations with several Env variants sharing conserved epitopes should guide the immune system towards the generation of bnAb responses^12^. The successive administration regimen allows the antibodies to gradually evolve to improve their recognition to the conserved components that are essential for viral function shared by diverse HIV-1 strains.

Thus, sequential immunizations with different variants of “native-like” HIV-1 Env that closely resemble the natural conformation of the Env spikes can potentially induce bnAb responses. Earlier, we have engineered HIV-1 chimeric Env (cEnv) into virus-like particles (VLPs) for a high-level of incorporation and enhancement of immunogenicity^13^. In the current study, we demonstrated that the sequential immunizations with a panel of Env-enriched VLPs from various HIV-1 clades elicited bnAb responses with significant breadth and potency of neutralization in rabbits.

## Results

### Env-enriched VLPs showed high levels of Env content and retained their physical and functional properties

**Figure 1:**
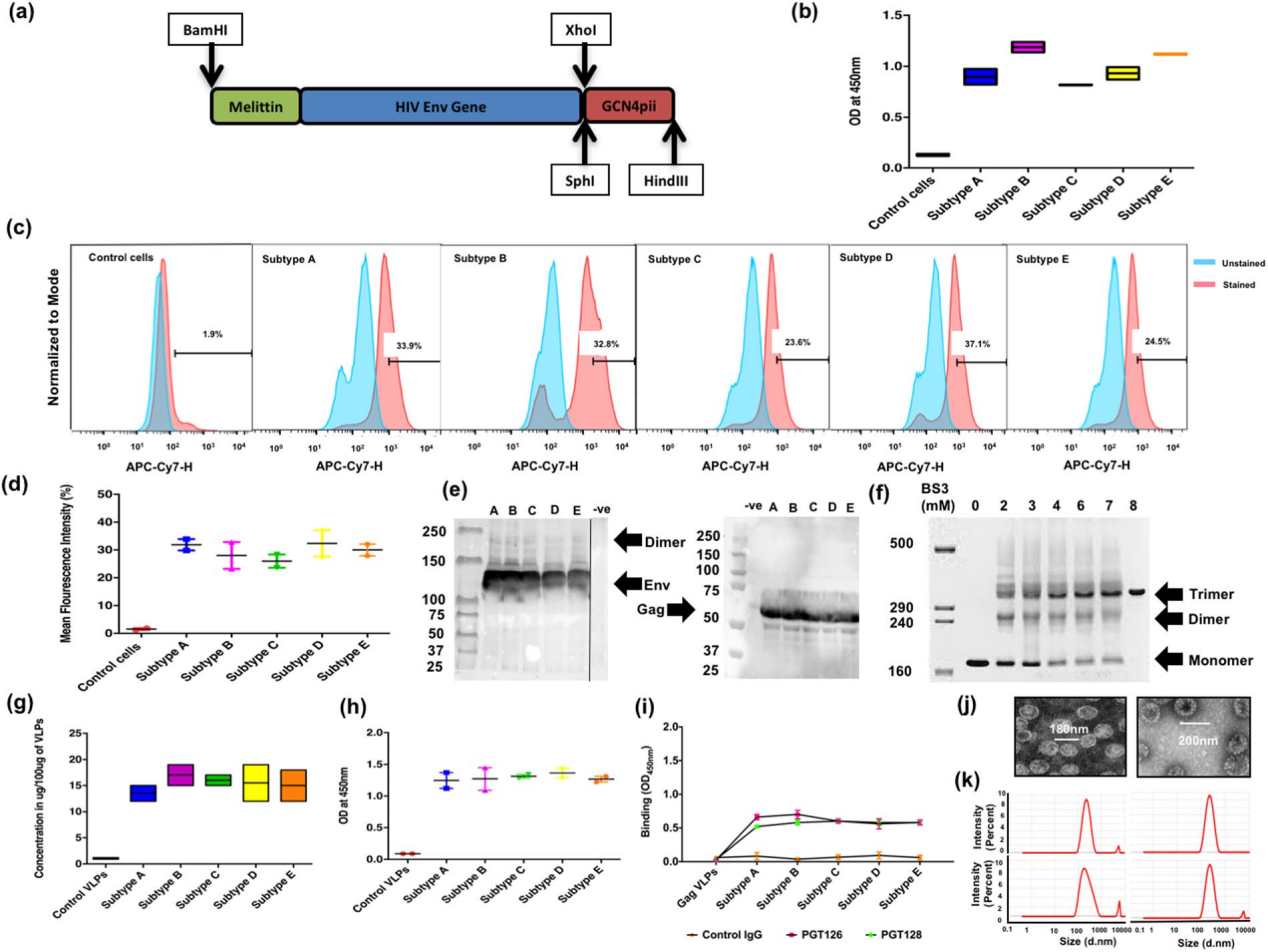
Chimeric HIV Env gene construct and characterization of VLPs. (**a**) Schematic representation of HIV-1 Env gene. The signal peptide-encoding sequences of HIV Env genes from subtypes A, B, C, D, and E were replaced by the honeybee melittin signal peptide sequence using overlapping PCR, to increase Env glycoprotein expression in SF9 insect cells. Conformation-stabilized trimeric Env proteins were made by the addition to C-terminus of a trimeric form of a leucine zipper sequence, GCN4pii to stabilize Env trimers. HIV Env gene containing melittin and GCN4pii were cloned into the pFastBac-1 transfer vector; (**b**) Surface expression of Env glycoprotein. The surface expression of Env glycoproteins on SF9 cells was determined by cell-based ELISA using polyclonal goat anti-HIV-1 Env followed by HRP-conjugated antibodies. The figure showed the data of three independent experiments and represented as mean ± SD; (**c**) FACS analysis of glycoprotein-CD4 binding. The amount of bound CD4 was analyzed by FACS using APC-Cy7 labeled goat anti-human CD4 antibody. An unstained and antibody isotype control cells stained with APC-Cy7 labeled anti-human CD4 antibody were used as negative control groups. Results in figure (**c**) showed one of the representative experiment, (**d**) showed the data of three independent experiments and represented as mean ± SD (n = 5); (**e**) SDS-PAGE analysis. Gag and Env glycoprotein profiles in VLPs were analyzed by SDS-PAGE in reducing conditions (in the presence of 1% BME). Unrelated (influenza) VLPs were used as a negative control group; (**f**) BS3 crosslinking assay. Prepared VLPs were crosslinked with BS3 at various concentration to confirm the oligomeric state of the HIV Envs as described in the materials and methods. (**g**) Total Env content in VLPs. A quantitative ELISA was done to determine the Env glycoprotein content in VLPs, using recombinant HIV-1 SF162 Env as a calibration standard. Unrelated (influenza) VLPs were used as negative control group. Results showed the data of three independent experiments and represented as mean ± SD; (**h**) PGT145 monoclonal antibody binding assay. The assay was performed as mentioned in material and methods. Unrelated (influenza) VLPs were used as negative control group. Results showed the data of three independent experiments and represented as mean ± SD; (**i**) HIV-1 Env VLPs binding to glycan-dependent PGT126 and PGT128. Unrelated antibody (anti-histidine) and Gag VLP were used as negative control groups in the assay. Results showed the data of three independent experiments and represented as mean ± SD; (**j**) TEM pictures of prepared VLPs; and (**k**) Zeta potential of the representative VLPs.

**Figure 2:**
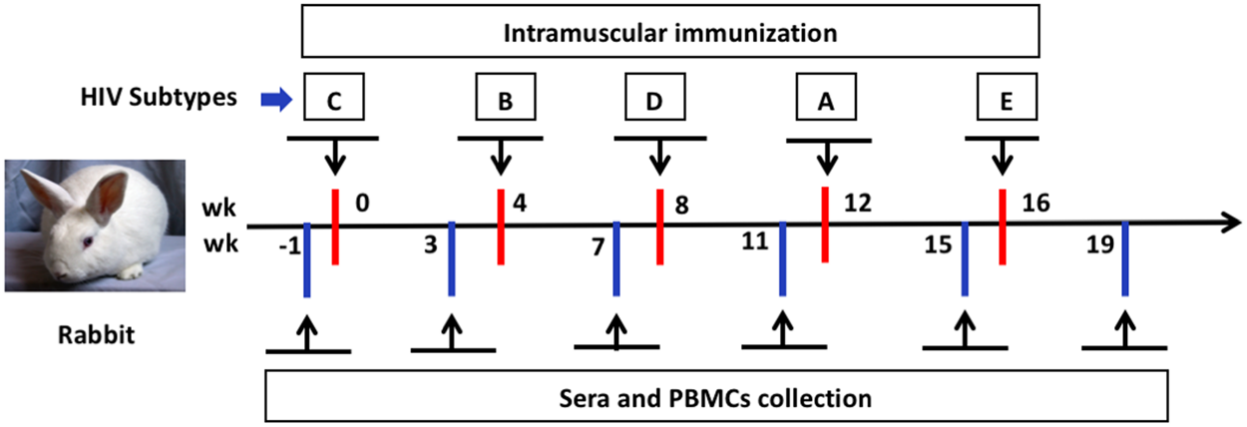
Figure represents the order of HIV Env VLPs used in the sequential-immunization strategy. Rabbits were immunized with PBS, Gag VLPs, single (subtype B) Env VLPs, a mixture of various Env VLPs, or sequential immunization of HIV Env VLPs through *i.m*. route of vaccination at week 0, 4, 8, 12, and 16 while the blood samples were collected at every 3^rd^ week after each vaccination.

Modified Env gene constructs were generated by replacing the original signal peptide encoding sequences with the honeybee melittin sequence and adding a trimeric form of leucine zipper sequence, GCN4pii to the C-termini of Env cytoplasmic sequences to increase Env glycoprotein production and to express conformation-stabilized trimeric Env proteins, respectively (Fig. 1a)^13^. In the current study, we have used Bac-to-Bac protein expression system in Spodoptera frugiperda (SF9) insect cells for the expression of various Env glycoproteins. An insect cell system is easy to handle, requires less maintenance, and economic without many of the potential bio-hazards associated with mammalian system. Insect cells have been used since the early years of HIV-1 vaccines^14,15^ and because these cells carry out many post-translational modifications including high-mannose type N- and O-linked glycosylations, resulting in glycoproteins with similar antigenicity and functionality as of mammalian system^16–18^. Cell-based ELISA results demonstrated that Env glycoproteins were expressed at the surfaces of recombinant baculovirus (rBV)-infected SF9 insect cells, as indicated by the enhanced OD_450_ value when compared to the control cells infected with unrelated (influenza) rBVs (Fig. 1b). We further tested the CD4 binding ability of expressed Envs on the SF9 cells using FACS^19^. An increase in the mean fluorescent intensities (MFIs) was found with cells infected with rBVs expressing specific Env versus unrelated (influenza) rBV infected control cells (Fig. 1c). The MFIs were 20–40% higher with cells infected by rBVs expressing various Envs than with control cells. We observed comparable CD4 binding efficiency with Env of different subtypes (Fig. 1d). The VLPs containing these Env and Gag were produced by co-infection of SF9 cells with rBVs expressing different Env (subtype A-E) and Gag at an optimal multiplicity of infection (MOI). The protein composition of VLPs was characterized by SDS-PAGE in the reducing conditions. Characteristic bands with molecular weights of 125–130 KD and 55–60 KD were detected for HIV-1 Env and Gag proteins, respectively. Comparable levels of Env and Gag proteins in three independent VLP preparations were demonstrated (Fig. 1e). Bis [sulfosuccinimidyl] suberate (BS3) cross-linking analysis showed that HIV Env VLPs primarily express the trimeric Env form (Fig. 1f). Quantitative ELISA results showed that the prepared VLPs contained a high level of Env (10–15 μg) per 100 μg of VLPs (Fig. 1g), which was comparable to previously observed incorporation levels^20^. In the PGT145 monoclonal antibody binding assay, prepared HIV-1 Env VLPs of various subtypes showed higher binding towards PGT145 antibody than the unrelated (influenza) VLP group. These results showed that Env in VLPs retained their conserved conformational structure (Fig. 1h). Insect cells and mammalian cells produce proteins with different glycosylation patterns. We analyzed the binding of PGT126 and PGT128 to HIV-1 Env VLPs produced in insect cells to assess if the Env retained the antigenicity as Env produced in a mammalian system. PGT126 and PGT128 antibodies bind specifically to the V3 glycan of HIV-1 gp120^21,22^. The binding results demonstrate that the insect cell prepared VLPs retain the Env antigenicity for these antibodies, which is implying the capability of the Env to induce antibody responses, specific to these glycan epitopes (Fig. 1i). Transmission electron micrograph (TEM) and Zetasizer data showed that the prepared VLPs were pure, spherical in morphology, and 180–200 nm in diameter (Fig. 1j and k). The data demonstrated that these stabilized Envs are incorporated into VLPs with their existing physical and functional properties. In the current study, we made five different HIV Env VLPs from each of the HIV-1 clades A, B, C, D, or E for sequential immunizations (Fig. 2). As mentioned in the Fig. 2, rabbits were sequentially immunized at specific time points with different vaccine formulations.

### Sequentially immunized animals showed higher serum reactivity towards the respective HIV-1 subtype Env (autologous) antigens

**Figure 3:**
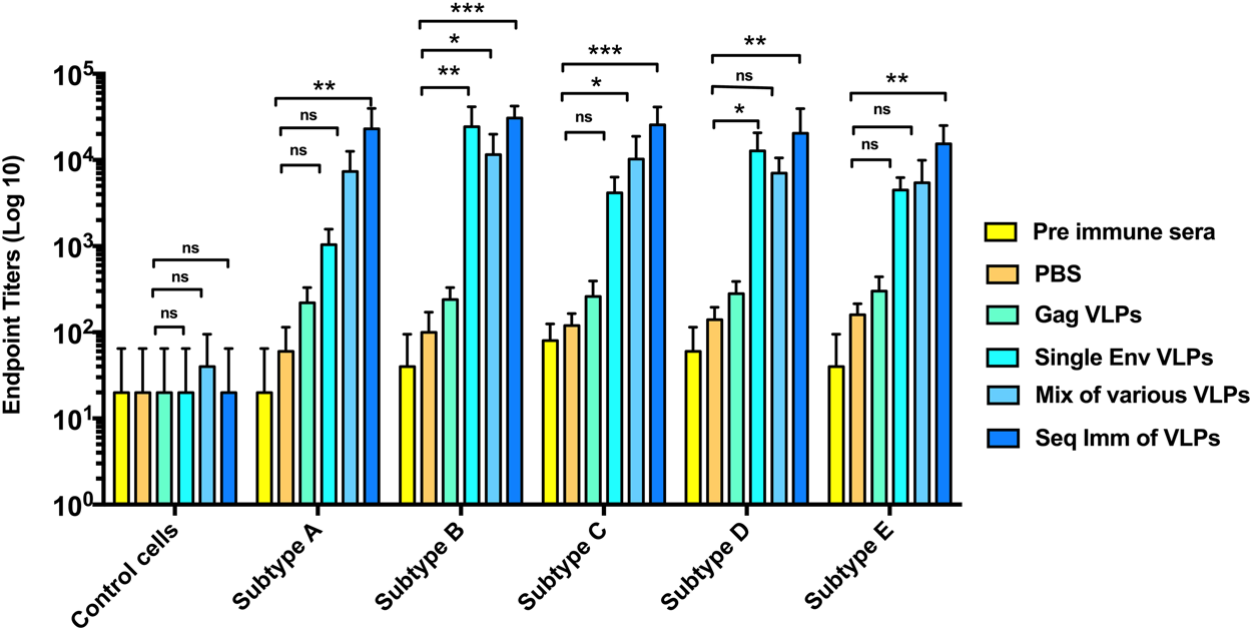
Endpoint titers against autologous Env. The figure demonstrates the results of cell-based ELISA to determine the endpoint titers against respective HIV Env (autologous) antigens as described in materials and methods. At the 3^rd^ week of last vaccination, the IgG endpoint titers were measured against HIV-1 Env of subtype A, subtype B, subtype C, subtype D, or subtype E expressed on HEK293T cells. Cells infected with unrelated plasmid and pre-immune sera have been used as negative control group. Rabbits were immunized with PBS, Gag VLPs, single (subtype B) Env VLPs, a mixture of various Env VLPs, and sequential immunization of HIV Env VLPs through *i.m*. route of vaccination. The highest dilution which gave an OD_450_ two-fold higher than that of the naïve group without dilution was designated as the antibody endpoint titer. Results showed the data of three independent experiments and results were expressed as the mean ± SD (n = 5).

An animal group was sequentially immunized with different HIV-1 Env VLPs through an intramuscular (*i.m*.) route at four-week intervals, and the results were compared with that of groups of animals immunized by repetitive immunizations of a mixture of various VLPs or a single type (subtype B) of Env VLPs. To provide additional insight into factors that might improve immunogenicity, we assessed serum reactivity towards the various HIV-1 subtype Envs after the last vaccination using cell-based ELISA. Results in Fig. 3 show IgG endpoint titers against Env antigens (autologous) from various HIV-1 subtypes, expressed on transfected cells. HKE293T cells transfected with an unrelated plasmid (control cells) and pre-immune sera were used as negative controls for the assay. In the sequential-immunization group, we observed that subtype-specific IgG endpoint titers were at its maximum. The sequential immunization group developed IgG with endpoint titers an order of magnitude greater (p < 0.001) than the mixture of various VLPs and other control groups. Simultaneously, single Env VLPs group demonstrated significantly (p < 0.01) higher sera reactivity towards subtype B Env antigen. On the other hand, the mixture of various VLPs group showed moderate levels of antibody levels towards each of the HIV-1 Env antigens than other negative control groups.

### Animals sequentially vaccinated with HIV-1 Env VLPs showed an increase in serum antibody levels towards the Con-S Env antigen

**Figure 4:**
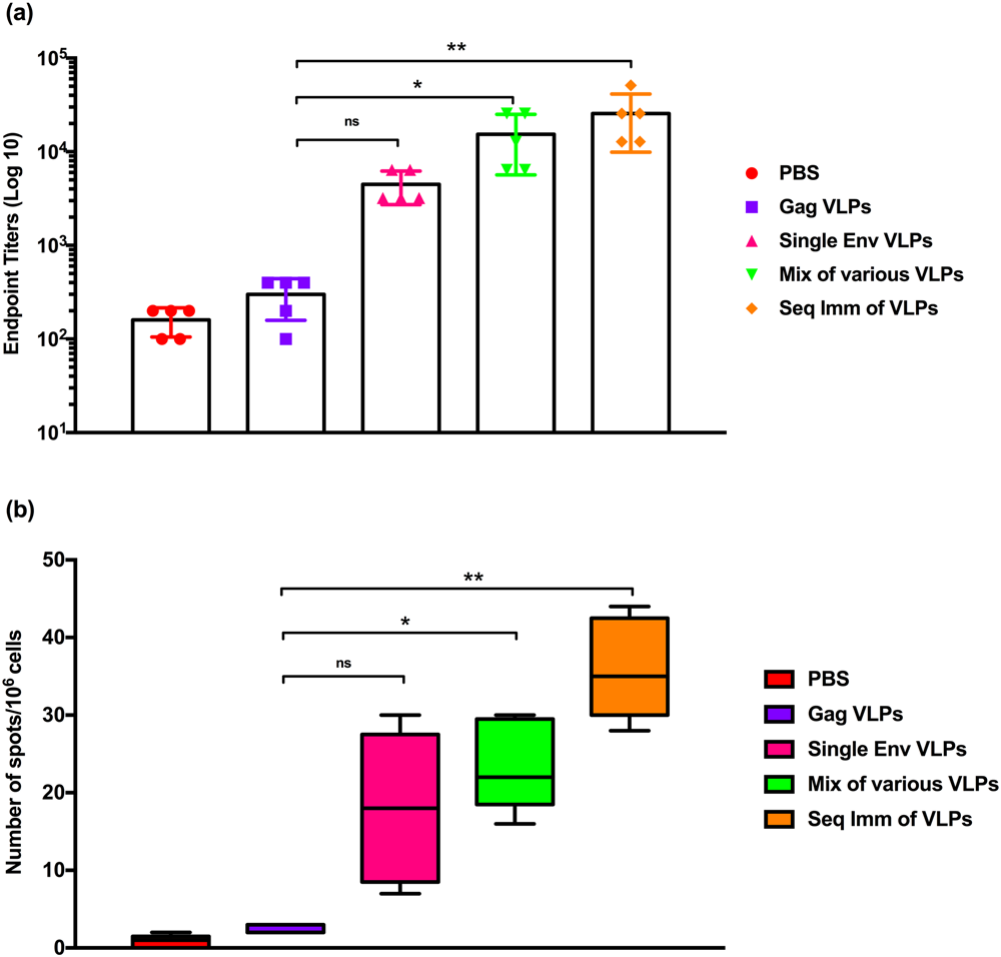
Con-S Env-specific antibody titers and IgGASCs. The figure represents antigen-specific (**a**) serum IgG endpoint titers; and (**b**) IgGASCs, at the 3^rd^ week of last vaccination using the recombinant Con-S Env protein as coating antigen. Rabbits were immunized with PBS, Gag VLPs, single (subtype B) Env VLPs, a mixture of various Env VLPs, and sequential immunization of HIV Env VLPs through *i.m*. route of vaccination. The highest dilution which gave an OD_450_ two-fold higher than that of the naïve group without dilution was designated as the antibody endpoint titer. Results showed the data of three independent experiments and results were expressed as the mean ± SD (n = 5). For ELISPOT, PBMCs from heparinized blood were collected and added to plates (1 × 10^6^ cells/well). Spots were developed as mentioned in materials and methods using capture/detection IgG antibody pair. Results were expressed as the mean ± SD (*n* = 5).

As the recent discovery of potent bnAbs from HIV infected individuals has galvanized interest in protective or therapeutic interventions^23^, it is important to acknowledge the role of antibodies specific to conserved epitopes in Env^24–26^. At the 3^rd^ week after the last vaccination, we determined serum IgG endpoint titers against HIV-1 Con-S Env protein by ELISA as described previously^27,28^. As shown in Fig. 4a, sequential immunizations with various VLPs induced significantly (p < 0.01) higher endpoint titers than those induced by other control treatments, when HIV-1 Con-S Env protein was used as the coating antigen.

### Sequential immunizations enhanced Con-S-specific IgG antibody-secreting cells (ASCs)

During the 3^rd^ week of the last vaccination, we evaluated IgGASCs specific to HIV-1 Con-S Env antigen using freshly prepared PBMCs from vaccinated animals by ELISPOT. We observed that the results were well correlated with antibody responses generated. ELISPOT data showed that antigen-specific IgGASCs were significantly (p < 0.01) higher in sequentially immunized animals than that of other animal groups. We observed 40–50 immunospots in the sequentially immunized group while the other groups developed only 20–25 spots. The number of detected spots was significantly (p < 0.01) higher (two-fold) in the sequential immunization groups than the mixture of various VLPs and single Env VLPs groups (Fig. 4b).

### Sequentially immunized animals showed antibodies with high avidity

**Figure 5:**
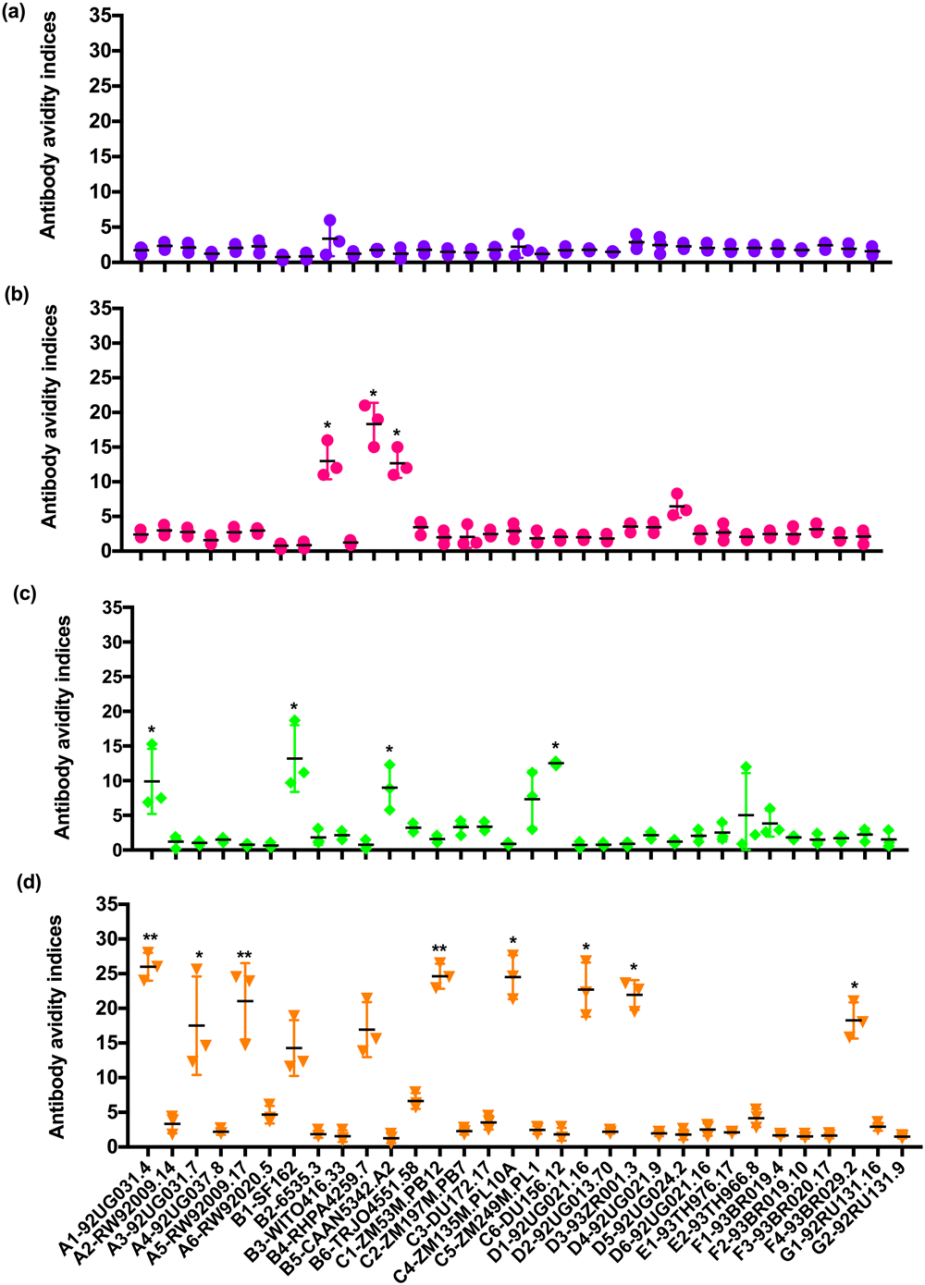
Avidity indices of immune serum against various pseudoviruses. Antibody avidity assays were conducted with immune sera collected at the 3^rd^ week of last vaccination from the animals immunized with (**a**) Gag VLPs; (**b**) single (subtype B) Env VLPs; (**c**) a mixture of various HIV Env VLPs; and (**d**) sequential immunization of HIV Env VLPs. The avidity index values were determined by measuring the resistance of antibody-envelope glycoprotein complexes in the ELISA by treatment with 1.5 M NaSCN. The avidity indices were calculated from the ratio of the absorbance value obtained with NaSCN treatment to that observed with PBS treatment multiplied by 100. Results were expressed as the mean ± SD (*n* = 5).

Several recent studies have shown a direct correlation between the avidity of neutralizing antibodies and HIV protective efficacy^29,30^. The results shown in Fig. 5 demonstrate that serum antibodies in the sequentially immunized group exhibited increased avidity compared to other immunization groups to the majority of pseudoviruses tested. Gag only VLP-immunized animals did not display an increase in avidity towards any of these pseudoviruses (Fig. 5a). Rabbits vaccinated with subtype B VLPs showed higher avidity towards the subtype B pseudoviruses especially B3, B5, and B6 (Fig. 5b). Intermediate enhancement of avidity indices to some of the strains, especially A1, B1, B5, and C6, were observed in the mixture of various VLP-immunized group (Fig. 5c). Serum antibodies from the sequentially immunized group showed increased avidity indices towards A1, A3, A5, A6, B1, B4, C1, C4, D1, and D3 HIV-1 pseudostrains (Fig. 5d). These results demonstrate that sequential-immunizations induced antibody responses with higher avidity indices to most of the pseudotyped virions tested including some of the tier 3 pseudostrains and might also indicate that sequential exposure of different Env sharing conserved epitopes to the immune system is important in triggering bnAb responses.

### Sequential immunizations with various HIV-1 Env VLPs enhanced bnAb responses

**Figure 6:**
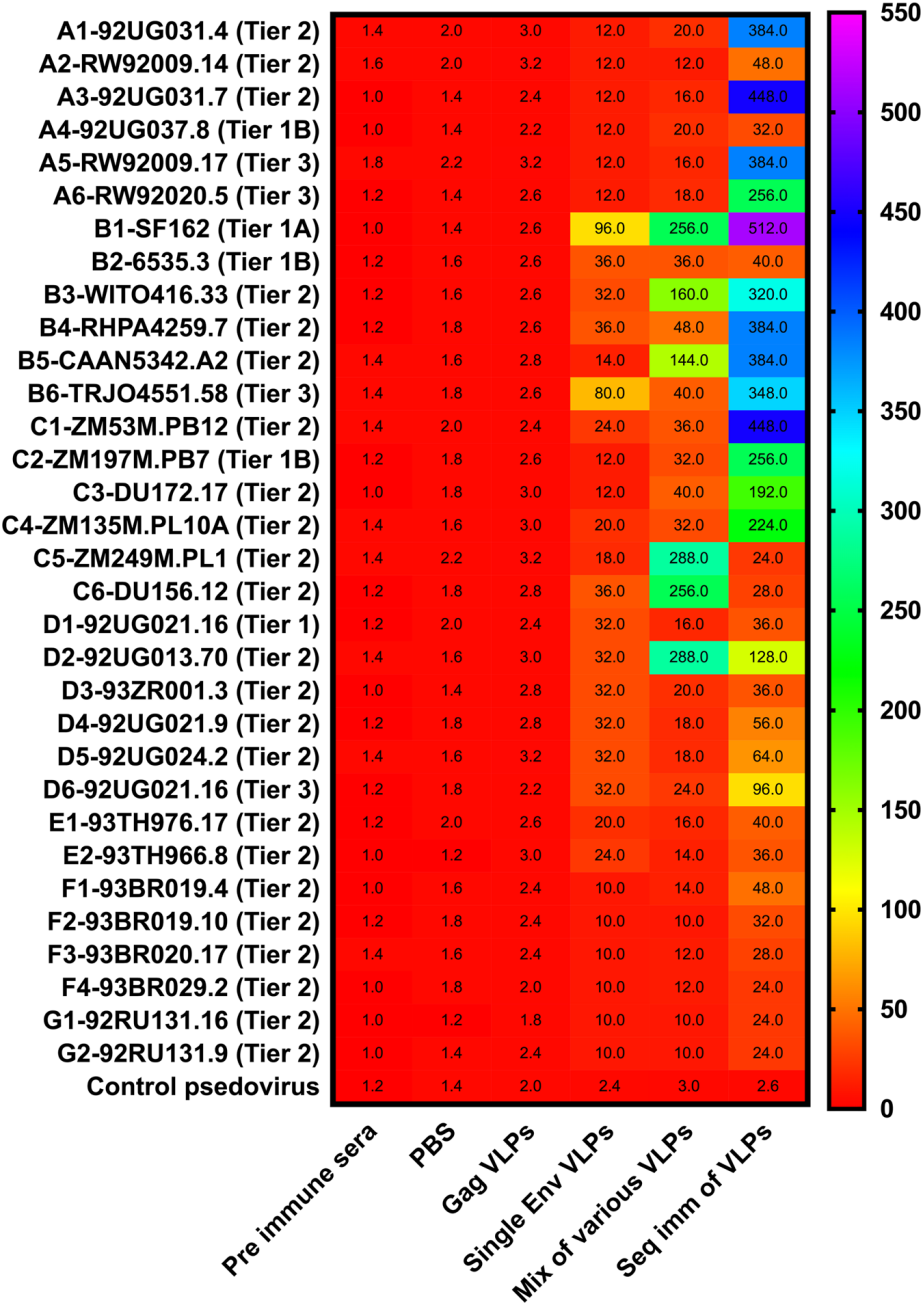
Sera neutralization assay. Heat map of ID_50_ values obtained with the sera tested individually against a panel of 32 pseudoviruses from tier 1, 2, and 3 of various HIV subtypes. The figure shows the ID_50_ values in the animal groups vaccinated with PBS, Gag VLPs, single (subtype B) Env VLPs, a mixture of various VLPs, and sequential immunization of Env VLPs. Results were compared with the ID_50_ values of the sera collected before vaccination (pre-immune sera), Gag only (non-Env) VLP, and unrelated (influenza H7N9) pseudovirus. The reciprocal of sera dilution necessary to achieve 50% neutralization was reported as the ID_50_ value. All values were calculated with respect to virus-only wells with the following formula: [(value for virus only minus value for cells only) minus (value for serum minus value for cells only)] divided by (value for virus minus value for cells only). Results showed the data of three independent experiments and results were expressed as the mean ± SD (n = 5).

Next, we evaluated neutralizing activity of the resulting immune sera against a panel of 32 HIV-1 pseudoviruses. In Fig. 6, the heat map shows the 50% inhibitory dilution (ID_50_), the reciprocal of sera dilution necessary to achieve 50% neutralization, from the various vaccine groups. Sera from the Gag VLP-immunized group did not show detectable ID_50_ (<10) against any of the pseudoviruses (data not shown). In the single Env VLP group, we observed ID_50_ of 150–200 especially against B1, B4, and B6. In the mixture of various VLPs immunization group, we observed ID_50_ in a range of 200–400 against B1, B3, B5, C5, C6, and D2 pseudoviruses, which represents a 22% (7 out of 32 pseudoviruses) neutralization potency. Low levels of ID_50_ (<20) against subtype E and F pseudoviruses were found while no detectable ID_50_ was observed against subtype G pseudoviruses. In sequentially immunized animals, we found significantly (p < 0.001) higher and broader ID_50_ values. Immune sera showed a significantly (p < 0.001) higher ID_50_ levels against all the pseudoviruses belonging to the subtypes A, B, and C (except A2, A4, B2, C5, and C6 pseudoviruses), especially A1, A3, A5, A6, B1, B4, B5, B6, and C1. Results showed that immune sera of the sequential immunization group neutralized ~70% of the pseudoviruses (23 out of 32 pseudoviruses). Interestingly, we have observed that the sequentially immunized group has shown greater ID_50_ values against some of the tier 3 HIV pseudostrains also such as A5, A6, and B6. Animals in this group showed ID_50_ in the range of 100–150 against subtype F and G pseudoviruses also, even though the vaccine formulation did not contain subtype F and G HIV-1 Env antigens. The negative control groups including pre-immune sera, non-Env (Gag) VLPs, and unrelated (influenza H7N9) pseudovirus showed no background neutralization. These data demonstrate the potential of sequential immunizations with various Env VLPs in inducing antibody responses with broad neutralizing activity when compared to the pre-immune sera or non-Env (Gag only VLPs) groups.

## Discussion

Major challenges for an HIV preventing vaccine that can elicit protective bnAb responses are the genetic diversity and mutability of HIV-1 target epitopes and the structural properties of the viral Env which hides conserved CD4 and co-receptor binding sites by modulating signature glycan motifs^31,32^. These challenges could be overcome by the design of novel Env immunogens that resemble the natural viral Env spikes and can trigger the selection and expansion of germline precursor and intermediate memory B cells to recapitulate B cell ontogenies associated with the generation of a bnAb response^32^. In the current study, we have engineered “native-like” HIV-1 Env antigens using GCN4pii trimerization motifs appended to full-length Env gene. Equally important for vaccine development is the identification of innovative vaccination strategies that can mimic the natural process of infection to drive somatic hypermutation and B cell maturation against heterologous primary virus envelopes^11^.

In the present approach, we have evaluated bnAb responses to HIV Env-enriched VLPs in rabbits, immunized through a sequential immunization pattern. We demonstrated that the sequential immunization of various Env VLPs induced bnAb responses at higher levels than the repetitive-homologous immunizations with a single Env VLP or a mixture of various VLPs. Antibodies induced by the mixture of various VLPs were different and reactive to only some of the variants. With the single Env VLP-vaccinated group, there was none of the antigenic diversity for the induction of bnAb responses. Many longitudinal studies of HIV-infected patients and SIV-infected macaques^33^ have demonstrated that the immune system gradually recognizes viral variants that emerge over time^34^. During the course of an infection, antibodies directed to Env undergo immunological maturation, increasing in avidity, conformational dependence, and neutralizing capacity^11^. We used diversified Env VLPs from various HIV-1 clades to generate bnAb responses both by presenting new epitopes as escape variants and by fostering the response against the more conserved epitopes. Our findings suggest that the sequential administrations of several Env VLPs could stimulate a stronger bnAb response than repetitive deliveries of a cocktail of these VLPs or single Env VLPs. A properly designed sequential-vaccination scheme with different variants of antigens offers hope to manipulate antibody development which might be able to more efficiently produce bnAbs^35,36^. As the development of bnAbs usually requires extensive antigen exposure over a long period of time, our sequential-immunization strategy included booster immunizations with one or more Env variants to shape the B cell immunity toward bnAbs responses^37,38^. Thus, this new analysis of optimal immunogen designs and our successful sequential-vaccination scheme provides some important novel insights into how immune responses to antigens develop and clues for creating a vaccine in the future.

Additionally, we observed that sequentially immunized animals also showed an increase in antibody avidity with a similar pattern as of ID_50_. Antibody avidity has been used as a measure of functional maturation of the humoral immune response^39–41^ and represents the combined binding affinities of a variety of antibodies and their multivalent antigen^42,43^. Although the role of high avidity in antibody-mediated neutralization of viruses has not been defined clearly, several recent studies have shown a direct correlation between the avidity of neutralizing antibodies and HIV protective efficacy^29,30^. Simultaneously, as most antigens have a diversity of antigenic determinants per protein molecule, an increased avidity could be a consequence of progressive appearance and accumulation of classes of antibodies, each specific for a distinctly different antigenic determinant^40^.

## Conclusion

Thus, a sequential-immunization approach was found to be much more potent in inducing broadly reactive antibody responses than repetitive immunizations of an antigen cocktail or a single Env antigen. From the above results, we concluded that a broader neutralization with higher avidity indices against various HIV pseudotyped virions was observed that represent strong bnAb responses in the sequentially immunized group of animals. Further efforts will be undertaken to engage germline B cell receptors and to stimulate B cell lineage development toward bnAbs targeting other vulnerable epitopes. Despite these encouraging results, it should be emphasized that these advances were made in low-bar *in vivo* models. It is therefore important to test the protective efficacy in non-human primates by SHIV challenges. Identifying vaccine delivery strategies and synergistic combinations of adjuvants that enhance the longevity and diversification of antibody responses is still largely an area of trial and error^44^.

## Materials and Methods

### Cells, reagents, and pseudoviruses

SF9 insect cells (ATCC, Manassas, VA, USA) (CRL-1711), and TZM-bl and HEK293T cells (ATCC, Manassas, VA, USA) (CRL-3216) were maintained in Sf-900 II media containing 1% Penicillin/Streptomycin and supplemented Dulbecco’s Modified Eagle’s Medium (DMEM), respectively. TZM-bl cells, HIV-1 Env clones of various strains, soluble human CD4, HIV-1 SF162 Env, Gag recombinant protein pr55, HIV-1 Con-S Env peptide pool, HIV-1 Con-S Env protein, goat anti-HIV-1 Env polyclonal antibody, and PGT126, PGT128, PGT145 antibodies were acquired from the NIH AIDS Reagent Program.

### HIV-1 Env gene constructs and VLPs preparation

Gene constructs and rBVs of HIV-1 Env (subtype A-E) proteins were generated as described previously^13^. We made five different rBVs using Env clones from each of the HIV-1 clades for sequential-immunizations: 92UG037.8 (subtype A), SF162 (subtype B), ZM53M.PB12 (subtype C), 92UG021.16 (subtype D), and 93TH976.17 (subtype E). These Env isolates were chosen on the basis of the homology of their amino acid sequences. The rBVs expressing HIV-1 Env glycoproteins from different subtypes or Gag protein were generated by using the Bac-to-Bac insect cell protein expression system (Life Technologies, Carlsbad, CA, USA)^45^. HIV-1 Env (Env/Gag) VLPs were produced by co-infection of SF9 cells with rBVs expressing trimeric Env and Gag protein at the optimum MOI. Gag VLPs produced by infection of SF9 cells with rBVs expressing Gag protein alone were used as a control. At 60 h post-infection, VLPs were concentrated from the cell culture supernatant by porous fiber filtration using ÄKTA Flux (GE Healthcare, Uppsala, Sweden) and purified using sucrose density gradient centrifugation as described previously^46^.

### Physical and functional characterization of prepared VLPs

To confirm the surface expression of Env glycoproteins, SF9 cells were infected with rBVs expressing Env at the optimum MOI. HIV-1 Env surface expression was determined by ELISA using goat anti-HIV-1 Env antibodies followed by horseradish peroxidase (HRP)-conjugated antibodies as described earlier^13^. Furthermore, the glycoprotein-CD4 binding capability was measured to examine whether the Env glycoprotein expressed on the cell surface were able to bind efficiently to soluble CD4 (5 μg/ml; human) by FACS using a FACSCanto II flow cytometer (Becton Dickinson, Franklin Lakes, NJ, USA)^47^. The VLP protein concentration was determined by Micro BCA protein assay (Thermo Fisher Scientific, Waltham, MA, USA) and contamination of endotoxins in VLPs was excluded by Limulus amebocyte lysate assay (Thermo Fisher Scientific, Waltham, MA, USA)^46^. Gag and Env glycoprotein profiles in VLPs were analyzed using SDS-PAGE in reducing conditions. Samples were heated to near boiling in the presence of a reducing agent; 1% β-mercaptoethanol (BME) (Pierce, Rockford, IL, USA) in addition to SDS, which further denatures the proteins and precludes the presence of aggregates by reducing disulfide linkages. We chemically cross-linked the freshly made VLPs with BS3 crosslinker (Pierce, Rockford, IL, USA) to confirm the oligomeric status of expressed protein in VLPs. Briefly, the prepared VLPs were incubated at room temperature in the presence of BS3 at different concentrations (final concentrations: 0, 2, 3, 4, 6, 7, and 8 mM, respectively) followed by SDS-PAGE in reducing conditions (1% BME)^48^. A quantitative sandwich ELISA was also done to determine total Env glycoprotein contents in VLPs, using HIV-1 SF162 Env as a calibration standard as described previously^47^. To check whether the expressed Env still retained their important natural conformation, we tested the binding of Env proteins towards conformational antibodies such as PGT145 monoclonal antibody as described earlier^49,50^ with minor modifications. Furthermore, we also examined the binding ability of prepared Env VLPs to glycan-dependent antibodies (PGT126 and PGT128; HIV-1 glycan binding bnAbs) by ELISA^21,22^. Unrelated IgG (anti-histidine) antibody and Gag VLP were used as negative control groups in the assay. The morphology, purity, size distribution, and zeta potential of prepared VLPs were determined by TEM (Zeiss, Oberkochen, Germany) and Zetasizer (Malvern, Massachusetts, MA, USA).

### Ethics statement

This study was carried out in strict accordance with the recommendations found in the Guide of the Care and Use of Laboratory Animals of the National Institutes of Health (NIH). All animal studies were approved by the Institutional Animal Care and Use Committee (IACUC) of Georgia State University under IACUC Number- A16023. 6–8-week-old, healthy female New Zealand white rabbits were purchased from Charles-River Laboratory (Wilmington, MA, USA), and housed in the University’s animal facility. Immunization and sample collection were performed under mild anesthesia that was induced and maintained with Acetylpromazine, and all efforts were made to minimize pain.

### Animals and immunization strategy

In this study, we have used groups of 5–6 New Zealand white rabbits for immunization because they generally exhibit relatively low background immune activity, can provide sufficient sera for various assays, and have also been extensively used in HIV-1 vaccine research^51^. As mentioned in Table 1, a sequential immunization regimen containing a panel of VLPs of various HIV-1 Envs (groups 5) was compared with repetitive-homologous immunizations of single (subtype B) Env VLPs (group 3) or a mixture of various Env VLPs (group 4). PBS (group 1) and Gag only VLPs (group 2) were used as negative control groups.

**Table 1:** Immunization groups.

Gps	Immunization 1	Immunization 2	Immunization 3	Immunization 4	Immunization 5
1	PBS	PBS	PBS	PBS	PBS
2	Gag VLPs	Gag VLPs	Gag VLPs	Gag VLPs	Gag VLPs
3	Subtype B- SF162 VLPs	Subtype B-SF162 VLPs	Subtype B-SF162 VLPs	Subtype B-SF162 VLPs	Subtype B-SF162 VLPs
4	Mix of various VLPs (A-E)	Mix of various VLPs (A-E)	Mix of various VLPs (A-E)	Mix of various VLPs (A-E)	Mix of various VLPs (A-E)
5	Subtype C- ZM53M.PB12 VLPs	Subtype B- SF162 VLPs	Subtype D- 92UG021.16 VLPs	Subtype A- 92UG037.8 VLPs	Subtype E- 93TH976.17 VLPs

All the vaccine formulations were given through *i.m*. route of immunization. The order of Env VLPs in the sequential immunizations was determined by the phylogenetic homology of the HIV-1 Env proteins in between different HIV-1 subtypes^52,53^. For immunizations, five doses of VLPs containing 100 μg of Env and 25 μg of Gag proteins were administered at week 0, 4, 8, 12, and 16 (Fig. 2). In the current study, we have used a dose of 100 μg of Env in total per rabbit in all the vaccination groups, including the mixture of various VLPs. In the mixture of various VLPs group, we have used 20 μg/animal of Env for each of the subtype (A/B/C/D/E).

### Samples collection and PBMCs isolation

Blood samples were collected for immune response assessments every 3^rd^ week after each vaccination. Blood samples were collected from the marginal ear vein of anesthetized rabbits and sera were collected from the clotted blood by centrifugation at 1500 × g for 10 min at 4 °C. PBMCs were isolated using Ficoll-paque PLUS (GE Healthcare Life Sciences, Pittsburgh, PA, USA) density gradient method as described earlier^54^.

### Endpoint titers against autologous and Con-S Env proteins

To determine the endpoint titers towards respective HIV Env (autologous) antigens at the 3^rd^ week of the last vaccination, a cell-based ELISA was performed as described in a previous study^55^. Briefly, HEK293T cells were transfected with various engineered HIV-1 Env plasmids with Lipofectamine 2000 (Invitrogen, Waltham, MA, USA). The transfected cells were seeded at a density of 5 × 10^4^ per well and later fixed by 80% acetone prior to serum inoculation. As conserved epitopes are the key for the generation of bnAb responses^24,25^, we also determined serum IgG endpoint titers by ELISA as described previously^27^, using recombinant HIV-1 Con-S Env protein (1 μg/ml) as the coating antigen. The highest dilution which gave an OD_450_ two-fold higher than that of the naïve group without dilution was determined as the endpoint titer.

### ELISPOT assay

For estimating Con-S-specific IgGASCs, multiscreen 96-well filtration plates (Millipore, Bedford, MA, USA) were coated with 1 μg/ml of recombinant HIV-1 Con-S Env protein and the freshly prepared PBMCs from individual vaccinated animal after last vaccination, at a concentration of 1 × 10^6^ cells/ml were added to each well (100 μl/well) and spots were developed using 3,3′-diaminobenzidine (DAB) (Pierce, Waltham, MA, USA) as described previously^46^ and counted by an ELISPOT reader (Biosys, Miami, FL, USA).

### Generation and titration of HIV-1 pseudoviruses

Immune sera neutralization breadth and potency and antibody avidity indices were evaluated using a highly sensitive, single-round pseudotype virus infectivity assay system. A total of 32 HIV-1 Env-pseudotyped virions from tier 1, 2, and 3 of various isolates (Table 2) were generated using the Fugene 6 transfection method (Promega, Madison, WI, USA). Pseudoviruses were produced by co-transfection of HEK293T cells with an Env-expressing plasmid of different clades and Env-deficient HIV-1 genomic backbone plasmid, pSG3ΔEnv.

**Table 2:** HIV-1 Env-pseudotyped virions from various clades.

S. No.	Name	Env Clone	Subtype	Co-receptor	Tiers	Location
1	**A1**	**92UG031.4**	**A**	**R5**	**Tier2**	**Uganda**
2	A2	RW92009.14	A	R5 and X4	Tier2	Rwanda
3	A3	92UG031.7	A	R5	Tier2	Uganda
4	A4	92UG037.8	A	R5	Tier1B	Uganda
5	A5	RW92009.17	A	R5	Tier3	Rwanda
6	A6	RW92020.5	A	R5	Tier3	Rwanda
7	**B1**	**SF162**	**B**	**R5**	**Tier1A**	**United States**
8	B2	6535.3	B	R5	Tier1B	United States
9	B3	WITO416.33	B	R5	Tier2	United States
10	B4	RHPA4259.7	B	R5	Tier2	United States
11	B5	CAAN5342.A2	B	R5	Tier2	United States
12	B6	TRJO4551.58	B	R5	Tier3	United States
13	**C1**	**ZM53M.PB12**	**C**	**R5**	**Tier2**	**Zambia**
14	C2	ZM197M.PB7	C	R5	Tier1B	Zambia
15	C3	DU172.17	C	R5 and X4	Tier2	South Africa
16	C4	ZM135M.PL10A	C	R5 and X4	Tier2	Zambia
17	C5	ZM249M.PL1	C	R5	Tier2	Zambia
18	C6	DU156.12	C	R5	Tier2	South Africa
19	**D1**	**92UG021.16**	**D**	**X4**	**Tier1**	**Uganda**
20	D2	92UG013.70	D	X4	Tier2	Uganda
21	D3	93ZR001.3	D	X4	Tier2	Zaire
22	D4	92UG021.9	D	X4	Tier2	Uganda
23	D5	92UG024.2	D	X4	Tier2	Uganda
24	D6	92UG021.16	D	X4	Tier3	Uganda
25	**E1**	**93TH976.17**	**E**	**R5**	**Tier2**	**Thailand**
26	E2	93TH966.8	E	R5	Tier2	Thailand
27	F1	93BR019.4	F	R5 and X4	Tier2	Brazil
28	F2	93BR019.10	F	X4	Tier2	Brazil
29	F3	93BR020.17	F	X4	Tier2	Brazil
30	F4	93BR029.2	F	X4	Tier2	Brazil
31	G1	92RU131.16	G	R5	Tier2	Russia
32	G2	92RU131.9	G	R5	Tier2	Russia

Pseudoviruses, generated for comparing the data of neutralizing antibody assay were produced by co-transfection of HEK293T cells with an Env-expressing plasmid of different clades and Env-deficient HIV-1 genomic backbone plasmid, pSG3ΔEnv using TMZ-bl neutralization assay.

At 72 h post-transfection, viral supernatants were harvested. Virus stocks were made cell free by low-speed centrifugation and filtration (0.45 μm) and later stored at −80 °C in growth medium containing 20% fetal bovine serum (FBS) (Thermo Fisher Scientific, Waltham, MA, USA). Pseudoviruses were further titrated for luciferase expression in the TZM-bl cells as described previously^56,57^ using Bright-Glo luciferase reagent (Promega Madison, WI, USA) and luminescence was measured using a Glomax Explorer luminometer (Promega Madison, WI, USA). Infection was measured by luciferase expression, and 200 × 50% tissue culture infective doses (TCID_50_) was calculated using Reed-Muench method^58^. These pseudoviruses exhibit a neutralization phenotype that is typical of most primary HIV-1 isolates^59,60^.

### Antibody avidity assay

The avidity indices of serum antibodies to Env proteins were determined by discriminating the weak binding in ELISA in the presence of 1.5 M sodium thiocyanate (NaSCN), as described previously^20^. To determine whether HIV Env VLPs induce antibody responses with enhanced antibody avidity towards 32 Env-pseudotyped viruses of various HIV clades were tested. Because pseudotyped virus-based neutralizing assays have been extensively used to evaluate an antibody’s capacity for blocking HIV infection, antibody avidity towards pseudovirus Env should reflect the antibody binding to HIV Env VLPs^28^. Pseudotype virions purified by filtration, pelleted and disrupted with 1% Triton X-100 were used as coating antigens. The avidity index values were determined by measuring the resistance of antibody-envelope glycoprotein complexes in the ELISA by treatment with 1.5 M NaSCN. The avidity indices were calculated from the ratio of the absorbance value obtained with 1.5 M NaSCN treatment to that observed with PBS treatment multiplied by 100.

### TZM-bl neutralization assay

The TZM-bl neutralization assay was used as previously described^61^ with some minor modifications. Two-fold serial dilutions of sera samples (starting from 1:10) from individual animal were plated in triplicate in 96-well flat bottom plate and 200 × TCID_50_ of each pseudovirus were added to the wells. Later, TZM-bl cells were added (1 × 10^4^/well in a 100 μl volume) in 10% DMEM growth medium containing DEAE-dextran (Sigma-Aldrich, St. Louis, MO, USA), at a final concentration of 7.5 μg/ml with appropriate negative and positive controls. Recombinant influenza H7N9 pseudovirus was produced as previously described^62,63^ by co-transfecting HEK293T cells with lentivirus vector pNL4–3-Luc R-E- (10 μg DNA) as backbone, pVKD-HA (5 μg DNA) and pVKD-NA (5 μg DNA), and used as negative viral control in the neutralization assay. Following a 48 h of incubation at 37 °C, 150 μl of culture medium was removed from each well and luminescence was measured as mentioned above. The reciprocal of sera dilution necessary to achieve 50% neutralization was reported as the ID_50_ value. All values were calculated with respect to virus-only wells with the following formula: [(value for virus only minus value for cells only) minus (value for serum minus value for cells only)] divided by (value for virus minus value for cells only)^28^.

### Statistical analysis

The data for antigen-specific IgG levels, IgG-ASCs, avidity indices, and ID_50_ were analyzed by two-tailed Mann-Whitney and Kruskal-Wallis tests. Statistical analysis of FACS data was done using SPSS version 12.0.1 for Windows. n = 5 rabbits per group and the results were expressed as mean ± standard deviation. Levels of significance (p-value) were compared between the sequential immunization group and other control groups. Tests were performed using GraphPad Prism 7 software (San Diego, California). p-values of < 0.05 (p < 0.05) were considered to be statistically significant. *p < 0.05; **p < 0.01, ***p < 0.001, ****p < 0.0001.
